# Search for the Pharmacophore of Histone Deacetylase Inhibitors Using Pharmacophore Query and Docking Study

**Published:** 2014

**Authors:** Atefeh Haji Agha Bozorgi, Afshin Zarghi

**Affiliations:** Department of Medicinal Chemistry, Faculty of Pharmacy, Shahid Beheshti University of Medical Sciences

**Keywords:** Histone deacetylase inhibitor, Cancer, Pharmacophor query

## Abstract

Histone deacetylase inhibitors have gained a great deal of attention recently for the treatment of cancers and inflammatory diseases. So design of new inhibitors is of great importance in pharmaceutical industries and labs. Creating pharmacophor models in order to design new molecules or search a library for finding lead compounds is of great interest. This approach reduces the overall cost associated with the discovery and development of a new drug. Here we elaborated an exact pharmacophore model for histone deacetylase inhibitors by using pharmacophore query and docking study. The data set used for the modelling exercise comprised of 383 molecules collated from the original literature. These molecules were used to crating the model and docking study was held with Zolinza, the recently FDA approved drug as potent histone deacetylase inhibitor. Our model consists of 5 features: Hydrogen bond donors, Hydrogen bond acceptors, H-bond donor/acceptors, Aromatic ring centers, and hydrophobic centers. With the aid of this pharmacophore model and docking result, 3D searches in large databases can be performed, leading to a significant enrichment of active analogs.

## Introduction

A pharmacophoreis the ensemble of steric and electronic features that is necessary to ensure the optimal supra molecular interactions with a specific biological target structure and to trigger (or to block) its biological response (1). The traditional medicinal chemistry definition of a pharmacophore is the minimum functionality a molecule has to contain in order to exhibit activity. Reports of the use of pharmacophore searching in three-dimensional databases in order to discover new lead compounds have described several different typesof query generation and search strategies (2). For example, the use of a training set approach, and receptor based approaches using either structural or pharmacophore databases have been reported (3,4). Searching 3D databases to discover novel activities of existing compounds has been widely applied in recent years. Two distinctly different approaches are being used: (i) shape-based methods, such as DOCK, (5) in which a protein structure is used to formulate the database query, to search for compounds whose structure complements the receptor’s steric characteristics; and (ii) pharmacophore- based methods, which search for compounds whose structure satisfies a certain pharmacophoric pattern, *i.e*. a specification of the geometric arrangement of a set of constraints formulated on a set of functional groups (6). In methods of the second type, one seldom sees detailed discussions of the problem of determining the pharmacophore, *i.e.* constructing an appropriate query of such a geometric arrangement of a set of functional-group constraints (7). Utilizing pharmacophore queries in large datasets in order to find new structures is of critical importance in last decade.Cancer is defined as a renegade system of growth that originates in human body. It is characterized, regardless of its type, by one common feature, which is unchecked growth that progresses toward limitless cell expansion. This process of unlimited proliferation is due to the fact that cells have lost their ability to undergo programmed suicide based on a process called apoptosis. According to the World Health Organization [WHO] statistics, cancer is considered the leading cause of death worldwide, responsible for 7.6 million deaths in 2008. Lung, stomach, liver, colon and breast cancers cause the highest numbers of cancer deaths each year, distributed between males and females. The rate of cancer incidence continues to increase, with around 13.0 million cases expected in 2030. In addition, it is expected that more than 70% of the casualties are from medium and low income countries. HDAC inhibitors can reactivate gene expression, and are potent inducers of growth arrest, differentiation, or apoptotic cell death in a variety of transformed cells in culture and in tumor bearing animals. Deregulation of HAT and HDAC has been implicated in the formation and development of certain human cancersby changing the expression pattern of various genes (8). It is therefore proposed that HDACs are a potential target for the development of small moleculeanticancer agent (9). HDAC inhibitors block the activity of the enzyme leading to the accumulation of acetylated histones. They alter the expression of 7–10% of genes and induce cell growth arrest, differentiation and/or apoptosis.A number of natural and synthetic HDAC inhibitors have been reported, and in recent years the importance of HDAC inhibitors has increased due to their efficacy against many malignant diseases (10). Several of these HDACinhibitors inhibit tumour growth and many of them are under clinical trials (11,12).The approval of SAHA (Zolinza®, Vorinostat) by the FDA for treatment of advanced cutaneous T-cell lymphoma (CTCL) has added histone deacetylase(HDAC) inhibitors to the clinician‘s armoury of anti-cancer therapeutics (13). The search for new drugs against cancer plays a central role in the research programs of pharmaceutical companies and many governmental organizations due to the impact of this disease. Histone deacetylase inhibitors can play an imperative role in the quest for new effective anticancer drugs and the design of new structures may be very beneficial (14). Here, we tried to find an exact pharmacophor model for histone deacetylase inhibitors intended for using in chemical structures in future.

## Experimental

***Methodology***

Dataset

383 molecules were collected from recently published literature papers (15-49) and the assay method was checked to be the same for all of them. The logarithm of measured IC_50_ values were used in pharmacophore modelling thus correlating the data linear to the free energy change. The two-dimensional (2D) chemical structures of the inhibitors were sketched using Hyperchem software and saved in pdb format. Subsequently, they were imported into MOE, converted into corresponding standard 3D structures and energy minimized to the closest local minimum using the molecular mechanics CHARMm force field implemented in MOE. The resulting 3D structures were utilized as starting conformers for MOE conformational analysis. Our database was created using the 3D structure of gathered molecules and their relevant IC_50_.

*Conformational analysis*

The molecular flexibilities of the collected compounds were taken into account by considering each compound as a collection of conformers representing different areas of the conformational space accessible to the molecule within a given energy range(50). Accordingly, the conformational space of each ligand was explored within MOE, which is basedon the generalized MMFF94x force field implemented in theprogram. Default parameters were employed in the conformationgeneration procedure, *i.e*., for each molecule, a conformationalensemble was generated with an energy threshold of 7 kcal/molfrom the local minimized structure that has the lowest energy level. A maximum of 250 conformers per molecule were generated.

*Pharmacophore query with MOE*

The PDQ (pharmacophore-derived query) strategy is based upon partitioning the compounds in a database of 3D structures in terms of the three to six center pharmacophore which are expressed by the compounds (51). These centers used in the pharmacophore were chosen to express groups commonly used in 3D searching and which represent important drug-receptor interactions: hydrogen-bond donor, hydrogen-bond acceptor, acid, base, aromatic center, and hydrophobic one; distance ranges covering most expected pharmacophore sizes were used (2-24 Å).We used MOE software (52) for generating pharmacophoric model of histone deacetylase inhibitors. Using MOE tools, a pharmacophoric model was created.

*Docking*

Molecular docking is a computer simulation procedure to predict the conformation of a receptor-ligand complex, where the receptor is usually a protein or a nucleic acid molecule (DNA or RNA) and the ligand is either a small molecule or another protein. It can also be defined as a simulation process where a ligand position is estimated in a predicted or pre-defined binding site. To identify possible binding modes for ligand in the subsequent analysis, a docking survey was initially pursued. For the docking study, the crystal structure of human histone deacetylase 8 complexed with Trichostatin A (PDB entry 1t64) was obtained from the Brookhaven Protein Data Bank. Water molecules were discarded from the pdb ﬁle and hydrogens were added. Charges were calculated by the Gasteiger–Huckel method. The structure was then subjected to energy minimization using the Powell method until an energy gradient of 0.05 kcal/mol was achieved. Zolinza was docked into the enzyme binding site using the Autodock 4 program (53). The binding site was initially deﬁned as all residues of the target within 10 Å from the zinc metal atom. From 10 independent genetic algorithm runs, at most 10 best ranked docking poses were obtained.

## Results

Combining pharmacophore query and docking study enabled us to create an exact pharmacophore model for histone deacetylase inhibitors. According to MOE results for pharmacophore query,the types of interaction centers are categorized as:

-Hydrogen bond donors

-Hydrogen bond acceptors

-Positive charge centers

-Aromatic ring centers

-Hydrophobic centers

**Figure 1:**
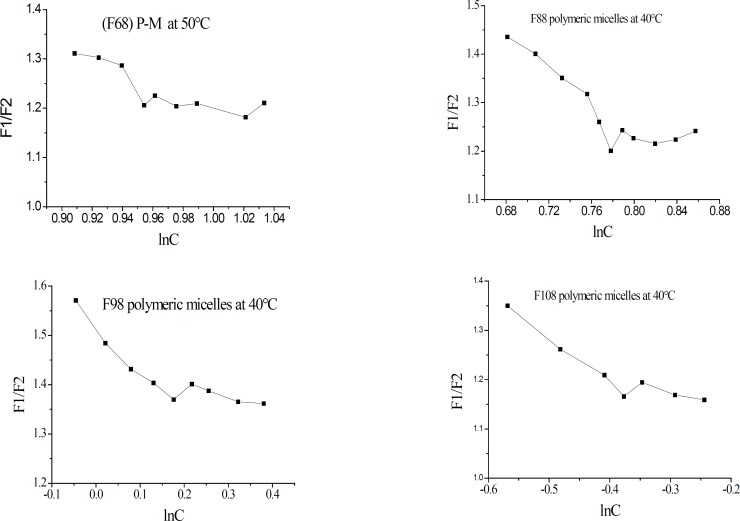
Pharmacophore model of histone deacetylase inhibitors. Orange: Aromatic center, Green: Hydrophobic area, Blue: H-bond acceptor, Violet: H-bond donor, Grey: H-bond donor/acceptorMeasured distance between h-bond donor and acceptor feature is about 11A˚. Mesured angel between cap and linker is about 110 ^o^.

In our obtained model, as shown in Figure 1, H-bond donor and acceptor, aromatic center and hydrophobic center is of great importance. Some of the critical distances between centers were measured and some angels were also mentioned in the model.

**Figure 2:**
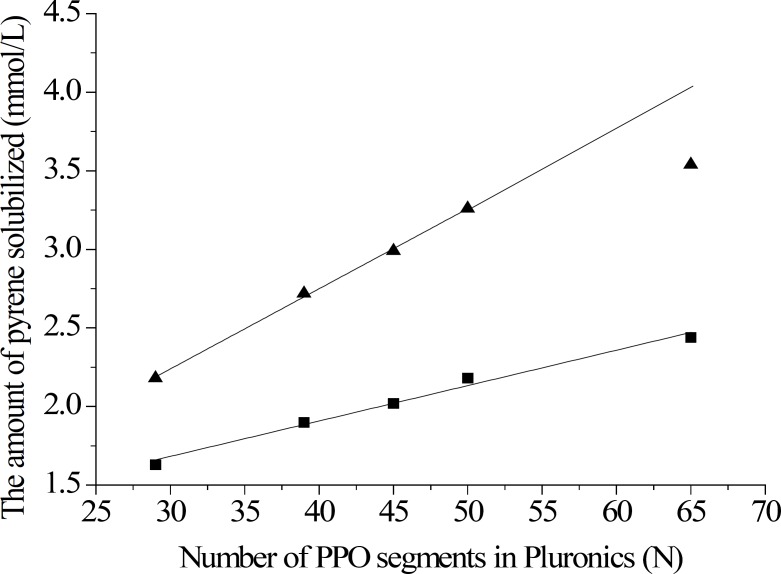
Docked Zolinza+ Obtained pharmacophor model. Orange: Aromatic center, Green: Hydrophobic area, Blue: H-bond acceptor, Violet: H-bond donor, Grey: H-bond donor/acceptor

Docking results, showed the binding mode of Zolinza in HDAC 8 active site. We used this conformation for validation of our pharmacophor model, by putting it in the obtained model (Figure 2).

1. Aromatic center, which is defined in Zolinza with a phenyl ring. As it shown, aromatic rings larger than one phenyl ring have enough space to be put here.

2. Hydrophobic center connected to aromatic moiety and inserted into a hydrophobic tube in active site. In Zolinza, the aliphatic chain plays this role.

3. H-Bond donor features, which in Zolinza, correlate with the oxygen of carbonyl groups. This group can be substituted with any other group which is capable of h-bond donation.

4. H-Bond acceptor groups, along the hydrophobic chain, which are in charge for hydrogen bond formation. NH groups in Zolinza take part in this task.

5. H-Bond donor/acceptor groups, proficient of hydrogen bond formation, either by donating hydrogen or by accepting one. OH of hydroxamide shows this ability in Zolinza.

## Discussion

The general pharmacophor for HDAC inhibitors is shown below:

**Figure:**
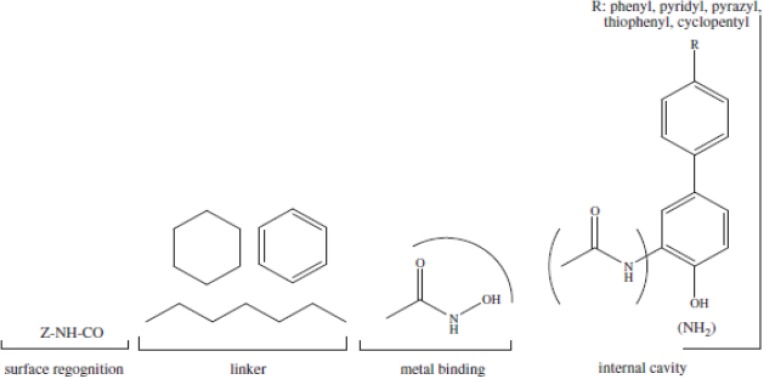


**Figure 3:**
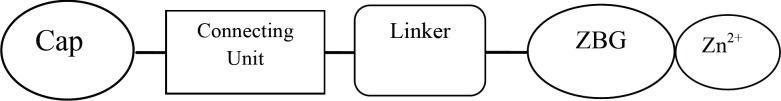
2D pharmacophor model for histone deacetylase inhibitors

 ZBG (the zinc binding group) chelating the zinc atom in the active site, a linker that accommodate the tubular access of the active site, and a cap group for interactions with the external surface, connected by a small connecting unit to the linker(54). According to our results, aromatic center, which entitled cap in other models, is better to be a phenyl ring or an indolyl group.Connecting unit should be capable of H-bond donation (NH group in Zolinza),so polar groups would be beneficial here (55). Hydrophobic linker, which is inserted into the narrow 11A˚deep hydrophobic channel which leads to the active site, generated by Phenylalanine, Histidine, Glycin, Methionin and Tyrosine.There is a 14A ˚ long internal cavity adjacent to the Zn-binding site, whereas the catalytic zinc ion is at the bottom of this channel. The internal cavity plays a significant role in the reaction that could potentially be exploited for drug development (55). Given the hydrophobic character of the internal cavity, non-polar substituents are preferred over polar groups such as pyrazyl, imidazolyl, pyridyl (Figure 3).

One of the three hydrophobic features was created complimentary to the Y306 which is important for the catalytic deacetylation mechanism. This HY feature enables Pi-Pi interaction between the phenyl rings of ligand and Tyrosine. The side chain of Tyrosine is involved in tunnel formation while its main chain makes the surface of the active site .A potent competitive HDAC inhibitor mimics the biological substrate, acetylated lysine, and coordinates with the catalytic zinc and thereby making the enzyme unavailable for the deacetylation(12). Zinc binding group, involves in H-bond formation, either by donating or accepting the hydrogen. Hydroxamide group can play this role better than other chelating groups. All hydroxamates were more active as HDACIs as compared with the corresponding carboxylic acids, probably due to the enhanced Zn (II) coordinating properties of the hydroxamates moiety (bidentate vs. monodentate binding) (55-57).
